# Co-Transplantation of Bone Marrow-MSCs and Myogenic Stem/Progenitor Cells from Adult Donors Improves Muscle Function of Patients with Duchenne Muscular Dystrophy

**DOI:** 10.3390/cells9051119

**Published:** 2020-04-30

**Authors:** Aleksandra Klimczak, Agnieszka Zimna, Agnieszka Malcher, Urszula Kozlowska, Katarzyna Futoma, Jaroslaw Czarnota, Pawel Kemnitz, Anna Bryl, Maciej Kurpisz

**Affiliations:** 1 Hirszfeld Institute of Immunology and Experimental Therapy of the Polish Academy of Sciences, Laboratory of Biology of Stem and Neoplastic Cells, 53-114 Wroclaw, Poland; urszula.kozlowska@hirszfeld.pl (U.K.); futoma.katarzyna@gmail.com (K.F.); 2 Institute of Human Genetics of the Polish Academy of Sciences, Department of Reproductive Biology and Stem Cells, 60-479 Poznan, Poland; agnieszka.zimna@igcz.poznan.pl (A.Z.); agnieszka.malcher@igcz.poznan.pl (A.M.); 3 Hospital MedPolonia, 60-693 Poznan, Poland; czarojaro@gmail.com (J.C.); 77551@interia.pl (P.K.); 4 Private Practice NeuroTeam, 60-539 Poznan, Poland; abryl.emg.poznan@gmail.com

**Keywords:** Duchenne muscular dystrophy (DMD), mesenchymal stem cells (MSCs), skeletal muscle stem/progenitor cells (SM-SPCs), cellular therapy of DMD, co-transplantation

## Abstract

Duchenne muscular dystrophy (DMD) is a genetic disorder associated with a progressive deficiency of dystrophin that leads to skeletal muscle degeneration. In this study, we tested the hypothesis that a co-transplantation of two stem/progenitor cell populations, namely bone marrow-derived mesenchymal stem cells (BM-MSCs) and skeletal muscle-derived stem/progenitor cells (SM-SPCs), directly into the dystrophic muscle can improve the skeletal muscle function of DMD patients. Three patients diagnosed with DMD, confirmed by the dystrophin gene mutation, were enrolled into a study approved by the local Bioethics Committee (no. 79/2015). Stem/progenitor cells collected from bone marrow and skeletal muscles of related healthy donors, based on HLA matched antigens, were expanded in a closed MC3 cell culture system. A simultaneous co-transplantation of BM-MSCs and SM-SPCs was performed directly into the biceps brachii (two patients) and gastrocnemius (one patient). During a six-month follow-up, the patients were examined with electromyography (EMG) and monitored for blood kinase creatine level. Muscle biopsies were examined with histology and assessed for dystrophin at the mRNA and protein level. A panel of 27 cytokines was analysed with multiplex ELISA. We did not observe any adverse effects after the intramuscular administration of cells. The efficacy of BM-MSC and SM-SPC application was confirmed through an EMG assessment by an increase in motor unit parameters, especially in terms of duration, amplitude range, area, and size index. The beneficial effect of cellular therapy was confirmed by a decrease in creatine kinase levels and a normalised profile of pro-inflammatory cytokines. BM-MSCs may support the pro-regenerative potential of SM-SPCs thanks to their trophic, paracrine, and immunomodulatory activity. Both applied cell populations may fuse with degenerating skeletal muscle fibres in situ, facilitating skeletal muscle recovery. However, further studies are required to optimise the dose and timing of stem/progenitor cell delivery.

## 1. Introduction

Duchenne muscular dystrophy (DMD) represents an X-linked genetic disorder, which may develop due to spontaneous or inherited mutations in the dystrophin gene, while the severity of the disorder is usually associated with a type of mutation [1,2]. A progressive deficiency of dystrophin, a protein responsible for the maintenance of skeletal muscle cell integrity and function, leads to a progressive loss of myofibres and reduces muscle volume and strength while substituting the degenerating muscles with fibrotic tissue and fat. Because of the genetic background of DMD, there is no satisfactory pharmacotherapeutic option to treat this progressive and devastating disorder. 

To date, different strategies involving the adoptive transfer of cells with the capacity for myogenic differentiation have been performed to treat DMD patients. However, the rate of success has been limited (reviewed by [3,4,5,6]). The cells most often applied for cellular therapies in DMD patients are myoblasts, myogenic precursor cells, or stem cells with the ability to differentiate into muscle cells. Pioneering clinical studies, performed over 25 years ago using myoblasts isolated from the skeletal muscles of healthy donors, were encouraging and indicated that a strategy could be developed to restore skeletal muscle function. However, limited positive results were reported [7,8,9,10,11]. The results that were inadequate to improve dystrophic muscle function using the transplanted myoblasts were explained by an inadequate number and restricted distribution of the transplanted cells in skeletal muscles, as myoblasts have a limited migratory capability and a relatively low proliferative potential [11,12]. Subsequent studies conducted by Skuk et al. using the ‘high density injection’ protocol for the intramuscular delivery of muscle precursor cells obtained from related healthy donors provided promising results in cell-based DMD treatment [13,14]. The therapeutic effect of individually-transplanted mesenchymal stem/stromal cells (MSCs) of bone marrow-origin (BM-MSCs) or stem/progenitor cells of myogenic-origin (SM-SPCs) was limited due to the complexity of muscular dystrophies and the biological activity of stem/progenitor cells. Moreover, muscle degeneration in DMD is accompanied by a chronic inflammation associated with the infiltration of immunocompetent cells involved in innate and adaptive immune responses, such as macrophages, subpopulations of T-cells CD4 and CD8, B-cells and dendritic cells [15,16]. Immunocompetent cells are a key cellular source of proinflammatory cytokines, and their presence in the dystrophic muscle affects the muscle fibre environment and, consequently, muscle repair and regeneration. The inflammatory response leads to an overexpression of proinflammatory cytokines and chemokines, including TNF-α, IFN-γ, IL-1, IL-6, and MCP-1, in the dystrophic muscle, and their systemic level reflects the stage of the disease [17]. Moreover, the inflammatory microenvironment of dystrophic muscles affects the efficacy of cellular therapy in terms of the survival and proliferation of the transplanted cells. The regulation of the innate and adaptive immune response may support muscle regeneration. This may be achieved through the immunomodulatory activity of MSCs to create a favourable environment for muscle stem/progenitor cell differentiation and muscle regeneration.

Understanding the key factors responsible for stable cell engraftment remains critical for a successful allogeneic or autologous stem/progenitor cell transplantation into a dystrophic muscle affected by an inflamed microenvironment. This may be accomplished by a clear biological characterisation of stem/progenitor cells conducted to improve the success rate of the cellular therapy of DMD. Based on the results of cellular therapies in DMD patients and the biological properties of BM-MSCs and SM-SPCs [18], we present the first observations on the effects of the co-transplantation of two populations of stem/progenitor cells obtained from adult healthy related donors, which provided an efficient improvement of skeletal muscle function in patients with DMD [16].

## 2. Materials and Methods

This experimental clinical procedure was approved by the local Bioethics Committee of the Poznan University of Medical Sciences, Poland (approval no. 79/2015). All patients or the patients’ parents gave their informed consent before the patients’ participation in the study. All procedures were performed in accordance with the principles of Good Clinical Practice (GCP) and the Declaration of Helsinki. All clinical procedures, such as tissue sample collection or cell delivery, were performed according to approved protocols. 

### 2.1. Patients and Donors

Three patients diagnosed with DMD between 11 and 22 years of age were enrolled into the study. All patients had an identified dystrophin gene mutation (Table 1). Bone marrow samples and skeletal muscle oligobiopsies were collected from three selected related healthy donors based on HLA matched antigens (Table 1). All samples were collected at the MedPolonia Hospital and transferred to the Institute of Human Genetics, Polish Academy of Sciences, Poznan, for stem/progenitor cell isolation. For Patients 1 and 3, the donor was the father, and for Patient 2, the donor was the patient’s sister, who was not a carrier of a dystrophin gene mutation. The HLA haplotype of the potential donor and recipients were determined for HLA class I and HLA class II antigens. The patients and selected donors were assessed for CMV, HIV, HBV, and HCV seropositivity. Cardiac and pulmonary functions were not affected in the enrolled patients. All patients were intellectually normal and were able to communicate with the physicians and hospital personnel.

### 2.2. Mesenchymal Stem/Stromal Cell Isolation and Expansion

MSCs were isolated from 50 mL of donor bone marrow (BM). Nucleated cell concentrates (NCCs) were obtained using the BioCUE BMA system (Biomet, Bridgend, UK,). The NCCs were resuspended in an alpha-MEM medium supplemented with 8% of human platelet lysate (both from Macopharma, Mouvaux, France) and antibiotics. The cells were expanded in a clinical-grade MSC production process using 636 cm^2^ closed MC3 cell culture systems (Macopharma, Mouvaux, France). Cells originating from BM were kept in standard in vitro culture conditions in a humidified atmosphere of 5% CO_2_, 37 °C. After 3–4 days of primary in vitro culture, the non-adherent cells were removed, and a fresh culture medium with supplements was added. The culture medium was changed every three days, up to 14–16 days after the start of culture, until the adherent cells reached a confluence of 70%–80%. The adherent cells were detached from the cell culture devices with an application of Acutase (P0) (BioLegend Inc., San Diego, CA, USA), conditioned in an alpha-MEM medium supplemented with 8% of platelet lysate, and placed into two new 1272 cm^2^ MC3 cell culture systems for expansion. The procedures were repeated up to P2 or P3 for MSC expansion (Table 2).

### 2.3. Isolation and Expansion of Skeletal Muscle Stem/Progenitor Cells (SM-SPCs) 

#### 2.3.1. Harvesting Muscle Biopsies

Skeletal muscle oligobiopsies were drawn from the same family donor (not a carrier of myopathic mutations) from whom BM was taken for MSC isolation. The procedure was conducted under local anaesthesia in aseptic conditions. Approximately 1 cm^3^ of skeletal muscle biopsy was harvested from the quadriceps, placed in a dedicated standard Hank’s medium, and transferred to the laboratory.

Tissue samples were also collected from the DMD patients (recipients of stem/progenitor cells) in order to verify the expression of the dystrophin gene, before and after the cellular intervention, using Real Time PCR. Muscle biopsies were performed with needle oligobiopsy and preserved in the RNALater buffer (Invitrogen, Thermo Scientific, Carlsbad, CA, USA) for subsequent isolation. 

#### 2.3.2. In Vitro Culture of SM-SPCs

Skeletal muscle oligobiopsies from the thigh region were mechanically dissected. Next, the tissues were subjected to digestion with a 0.02% collagenase solution (Sigma-Aldrich, St. Louis, MO, USA). The obtained cell suspension was briefly filtered through an 80 μm mesh, centrifuged and plated on a gelatine-covered culture dish. The myogenic stem/progenitor cells were cultured with 4.5 g/L of glucose DMEM supplemented with a 20% foetal bovine serum, 1% Glutamax, 1% antibiotic and antimycotic solution, 5 ng/mL of bFGF (Sigma-Aldrich, St. Louis, MO, USA) and maintained in standard in vitro conditions: 5% CO_2_, 37 °C and 95% humidity. To achieve large-scale cell preparation, the SM-SPCs were passaged every 4–5 days, and the medium was replaced every other day. After reaching 500–600 million cells (after 4–5 weeks), the cells were washed several times in a DMEM medium w/o phenol red and then resuspended in 3–5 mL of a 0.9% NaCl solution with a 5% patient serum and/or 1% human serum albumin and administered to the patient.

### 2.4. Transplantation of Mesenchymal Stem Cells and Myogenic Stem/Progenitor Cells 

Before the BM-MSC and SM-SPC transplantation, both populations of cells were resuspended in 5% of the autologous serum of the recipient. A mixed population of MSCs and myogenic stem/progenitor cells was transplanted into a selected dystrophic muscle using the ‘high density injection’ protocol introduced by Skuk et al. [13]. Cells were delivered under general anaesthesia to avoid undesired stress associated with multiple injections, especially in the minor patients. The injected area was cleaned and disinfected with chlorhexidine gluconate in isopropanol. To control the density of cell injection, an OpSite sterile transparent dressing with a 5-mm grid was used (Smith & Nephew, Watford, UK). The injections were performed with a 250 µL Hamilton syringe attached to a repeating dispenser (Hamilton, Reno, NV, USA). A volume of 10 µL of cell suspension was delivered at each trajectory. The quantitative details of the injected area and number of cells delivered to each patient are listed in Table 1.

### 2.5. Immunosuppression

A short-time immunosuppression using tacrolimus administration with an initial dose of 0.3 mg/kg/day started on day 7 before the delivery of BM-MSCs and myogenic stem/progenitor cells. After 4 weeks, tacrolimus was withdrawn, and at the end point of immunosuppression, its level in the peripheral blood was assessed as ranging from 4.2 to 7.8 ng/mL. The patients were monitored for blood morphology, haematocrit, lipid profile, level of glucose in the serum and creatine kinase (CK). 

### 2.6. Characteristics of Isolated BM-MSCs and Myogenic Stem/Progenitor Cells

The phenotypes of the cultured BM-MSCs and myogenic progenitor cells were characterised using flow cytometry with monoclonal antibodies specific for: HLA-DR (FITC, YE2/36-HLK) and HLA-ABC (Alexa Fluor 647 nm, W6/32) (both from Bio-Rad, Hercules, CA, USA) and: CD105 (PE, 226), CD73 (APC, AD2), CD90 (FITC, 5E10), CD146 (PE, P1H12), CD31 (FITC, WM59), CD45 (APC, HI30) and CD34 (FITC, 581) (all from BD Pharmingen, Franklin Lakes, NJ, USA) and for costimulatory molecules: CD154 (FITC, TRAP-1), CD40 (FITC, 5C3), CD80 (FITC, L307.4) and CD28 (FITC, CD28.2) (all from BD Pharmingen, Franklin Lakes, NJ, USA). Antibodies for: IgG1 κ (FITC), IgG1 κ (PE), and IgG1 κ (APC) (all from BD Pharmingen, Franklin Lakes, NJ, USA) were used as isotypic controls. The analyses were performed with an Amnis^®^ Imaging Flow Cytometers (Merck Millipore, Kenilworth, NJ, USA) device and analysed using the IDEAS Application software v 6.0 (Amnis part of EMD Millipore, Seattle, WA, USA)

### 2.7. Assessment of the Spontaneous Fusion of BM-MSCs and SM-SPCs In Vitro

To assess the ability of BM-MSCs and SK-SPCs to fuse in vitro, 0.5 x 10^6^ cells of each population were resuspended in serum-free media and stained with PKH26 (red) or PKH67 (green) (both from Sigma-Aldrich, St. Louis, MO, USA), respectively, by adding 2 µL of proper PKH diluted in 250 µl of diluent C in a cell pellet. The cells were incubated for 3 min at RT, after which the reaction was interrupted by adding 1 mL of 1% BSA (Abcam, Cambridge, UK). The cells were washed 3 times and resuspended in the culture medium, and 1 × 10^4^ of PKH67-stained muscle stem/progenitor cells and 1 × 10^4^ of PKH26-stained BM-MSCs originating from the same donor were placed on a 24-well plate in duplicates (a total of 2 × 10^4^ cells per well). Co-culture of BM-MSCs labelled with PKH-26 (red) and SM-SPCs labelled with PKH-67 (green) was performed in a DMEM F12 medium supplemented with 10% FBS, 1% L-glutamine and 1% of antibiotic/antimycotic solution. The fusion was monitored, and images were acquired every day. On day 7, the cells were detached from the plate, fixed and permeabilised in the BD cytoperm/cytofix agent (BD Biosciences, San Jose, CA, USA), stained for 20 min with DAPI (Vecta Shield), and analysed with the Amnis^®^ Imaging Flow Cytometers (Merck Millipore, Kenilworth, NJ, USA) system.

### 2.8. Analysis of Histopathology and Local and Systemic Immune Responses

Muscle biopsies were taken on day 0 prior to the BM-MSC and SM-SPC application, four weeks and six months after the completion of cellular therapy. Tissue samples were fixed in 10% formalin and stained with haematoxylin and eosin (H + E) for the histopathological assessment. Subsequently, immunostaining for dystrophin detection was performed using the anti-dystrophin polyclonal antibody PA5-16734 diluted 1:400 (Invitrogen, Rockford, IL, USA), dedicated for paraffin-embedded sections. After the deparaffinisation and re-hydration, heat-induced epitope retrieval was performed in the citrate buffer pH = 6.0 for 20 min at 97 °C. Next, the slides were incubated with the primary antibody overnight at 4 °C. After incubation, the slides were washed in the TRIS buffer, and the binding of the primary antibody was visualised using the EnVision^TM^ FLEX/HRP detection system with Magenta Substrate Chromogen (Dako, Glostrup, Denmark) according to the manufacture’s guidelines. As a positive control, a skeletal muscle specimen from non-DMD individuals taken during autopsy was used (approved by the local Bioethics committee (KB 746/2012)).

The systemic immune response was assessed using a panel of 27 cytokines and chemokines in the Bio-Plex Pro^TM^ Human Cytokine GRP I Panel 27-Plex and Multiplex ELISA system (BioRad, Hercules, CA, USA) according to the manufacturer’s protocol. All reagents necessary to perform the ELISA-array analysis were included in the Bio-Plex Pro^TM^ Human Cytokine GRP I Panel 27-Plex kit. Serum samples of patients were collected during the out-patient visits planned for weeks 2, 4, 8, 12, and 24 after the cellular therapy and stored at −80 °C before use. Prior to the analysis, the serum samples were diluted 1:4 (v/v) with the diluent included in the kit, as recommended in the Bio-Plex Cytokine Assay Instruction Manual (Bio Rad, Hercules, CA, USA) and analysed in triplicates. The following cytokines and chemokines were included in the analysed panel: IL-1β, IL-1Ra, IL-2, IL-4, IL-5, IL-6, IL-7, IL-8, IL-9, IL-10, IL-12(p70), IL-13, IL-15, IL-17A, IFNγ, IFNγ-inducible protein (IP10), TNFα, monocyte chemoattractant protein (MCP)-1, macrophage inflammatory protein (MIP)-1α, MIP-1β, vascular endothelial growth factor (VEGF), platelet-derived growth factor PDGF-β, granulocyte-macrophage colony stimulating factor (GM-CSF), granulocyte colony stimulating factor (G-CSF), Eotaxin, and RANTES. The results of the analysis of the cytokines and growth factors present in the patients’ sera were compared to the control sera taken from the three healthy volunteers. The analysis was performed using the Bio-Plex 200 system (Bio-Rad, Hercules, CA, USA) with the Bio-Plex Manager^TM^ Software 6.1 (Bio-Rad Laboratories Inc., Hercules, CA, USA). 

### 2.9. RNA Extraction and Real-Time PCR

Total RNA was extracted from the tissue samples collected from DMD patients according to the manufacturer’s protocol of Tri^®^Reagent (Sigma-Aldrich, St. Louis, USA). The RNA samples were then purified using a Turbo DNase Kit (Invitrogen, Thermo Scientific, Carlsbad, CA, USA). Reverse transcription reactions were performed in standard conditions using Superscript Reverse Transcriptase IV (Invitrogen, Thermo Scientific, Carlsbad, CA, USA). Real-time PCR was performed using a SsoAdvanced Universal SYBR Green Supermix (BioRad, Hercules, CA, USA). 

The threshold cycle (Ct) values of each studied transcript were analysed with the CFX ConnextTM Real-Time System (Bio-Rad Laboratories, Hercules, CA, USA). All samples were run in triplicates. The relative level of the dystrophin gene expression was normalised to three housekeeping genes (*ACTB*, *TBP* and *GAPDH*) using the GeNorme algorithm. Validated primers (PrimePCR™ PCR Primers), listed below (Table 3), were purchased from Bio-Rad Laboratories.

### 2.10. Electromyography Examination (EMG)

The quantitative EMG examination was carried out by analysing motor unit potentials (MUP) using the Multi-MUP Analysis software version 2.10 (Keypoint, Natus, Alpine Biomed, Skovlunde, Denmark) applied with the Keypoint device (Keypoint, Natus, Alpine Biomed, Skovlunde, Denmark). The procedure was performed with a template corresponding to the decomposition technique according to Stalberg [19]. The activity of the biceps brachii muscles was recorded using a concentric needle electrode. The MUPs were recorded at up to 20 MUPs per examination and calculated using the multi-MUP algorithm. Ambiguous MUPs were rejected. The MUPs were analysed for amplitude, duration, area and size index.

### 2.11. Statistical Analysis

All graphics were prepared and statistical analyses were performed using the GraphPad Prism version 5 software (GraphPad Software Inc., San Diego, CA, USA). The double-tailed *T*-test for unpaired samples, with Welch’s correction for unequal variances, 95% confidence, was used to assess the multiplex ELISA *p*-value. The *p*-value of the qPCR results was evaluated using the one-way ANOVA nonparametric test.

## 3. Results 

### 3.1. Clinical Outcome of the Applied Protocol

The patients were monitored in an outpatient clinic over a period of six months (two, four, and eight weeks, three and six months).

#### 3.1.1. Patient 1

**Figure 1:**
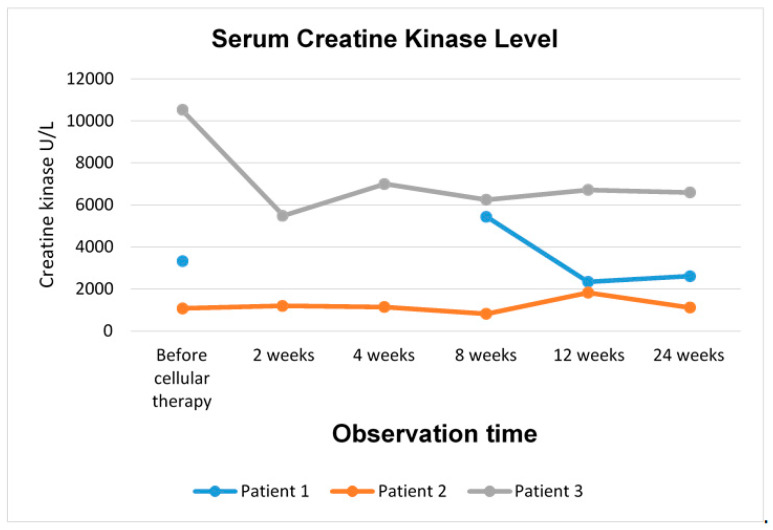
Creatine kinase level in DMD patients after cellular therapy with BM-MSCs and myogenic stem/progenitor cells.

Patient 1 received a total of 820 × 10^6^ cells of a mixed population of MSCs and myogenic progenitors into the right gastrocnemius over the surface of 18 cm^2^. There were no complications after the cell delivery, no fever or other adverse effects, and 24 h after the procedure was completed, the patient was discharged from the hospital. Slight erythema on the site of cell delivery was visible during the next few days, after which the skin returned to normal appearance. Four weeks after the cell delivery, blood morphology, haematocrit, lipid profile, glucose, sodium, potassium, and chlorides were at normal levels. Six months after the cellular therapy, kinase creatine level was assessed at 2613 U/L, compared to the initial level of 3330 U/L before the cellular therapy (Figure 1).

#### 3.1.2. Functional Assessment

Prior to the cellular therapy, a better mobility of the left lower limb was diagnosed. Six months after the cell delivery, a weak contraction allowing movement was observed in the right limb. On the right side, Patient 1 was able to keep the lower limb bent when placed on the ground better than the left one. The patient lifted their right limb better primarily in the hip joint (the left foot was raised to a height of about 20 cm). Superficial and deep feeling of the lower limbs were correct. EMG assessment for Patient 1 was impossible to interpret due to a lack of cooperation between the patient and the physician. 

#### 3.1.3. Patient 2

Patient 2 was treated with a total of 740 × 10^6^ cells of a mixed population of MSCs and myogenic stem/progenitor cells administered into the right biceps brachii over the surface of 10 cm^2^. There were no adverse effects after the cell delivery, and 24 h after the procedure was completed, the patient was discharged from the hospital. 

Initially, kinase creatine level amounted to 1078 U/L; two months after the cell delivery, it decreased to 824 U/L, and after six months, it increased to 1121 U/L (Figure 1). 

**Figure 2:**
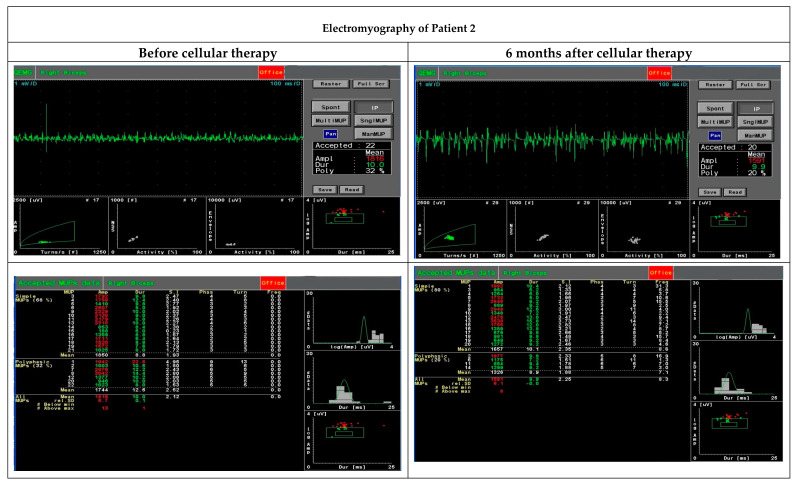
Functional assessment performed with electromyography before and six months after cellular therapy in Patient 2 revealed an improvement in motor unit parameters, especially area and size index.

Functional assessment performed with EMG six months after the cellular therapy revealed an improvement in motor unit parameters, especially area and size index, compared to the initial assessment before the administration of MSCs and myogenic progenitors (Table 4, Figure 2). 

#### 3.1.4. Patient 3

Patient 3 was treated with a total of 700 × 10^6^ cells of a mixed population of MSCs and myogenic progenitors administered into the right biceps brachii over the surface of 9 cm^2^. After the cell transplantation, a swelling of the right shoulder muscles was observed during the first few days, and a pain was reported two and four weeks after the cell injection. No adverse effects were observed two months after the cell transplantation. Three weeks after the transplantation, serum lipids were elevated (258 mg/dL), and creatinine level decreased (0.22 mg/dL). Blood morphology, haematocrit, glucose, sodium and potassium levels were normal.

Kinase creatine level was monitored. Before the cellular therapy, it was assessed at 10,528 U/L, and during the follow-up period, it decreased to 6600 U/L six months after the cell transplantation (Figure 1).

**Figure 3:**
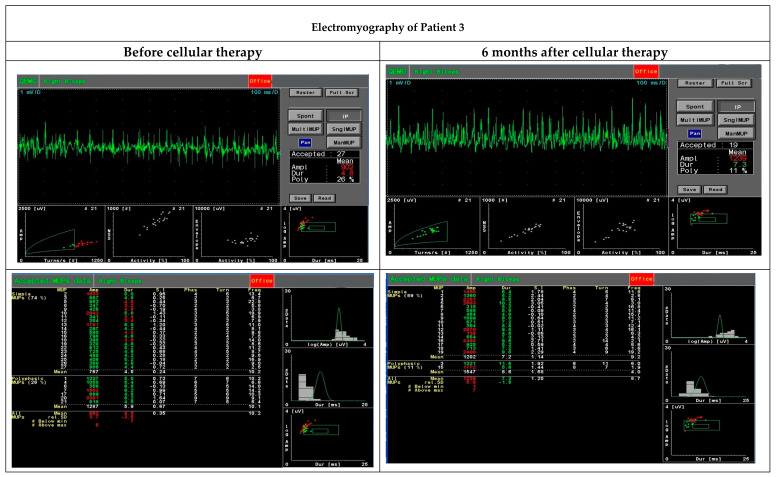
Functional assessment performed with electromyography before and six months after cellular therapy in Patient 3 revealed an increase in motor unit parameters, especially duration, amplitude range, area and size index.

An intramuscular EMG study performed six months after the cellular therapy observed an increase in motor unit parameters, especially duration, amplitude range, area and size index, compared to the initial test before the administration of MSCs and myogenic stem/progenitor cells (Table 5, Figure 3). These parameters were recognised as myopathic.

### 3.2. Common Phenotype of BM-MSCs and Myogenic Stem/Progenitor Cells

**Figure 4:**
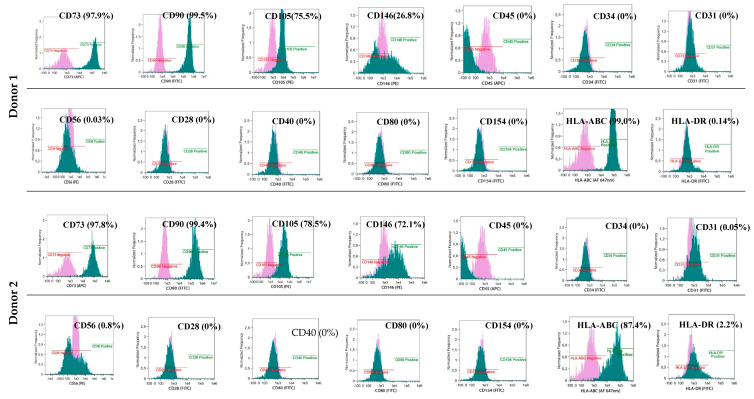

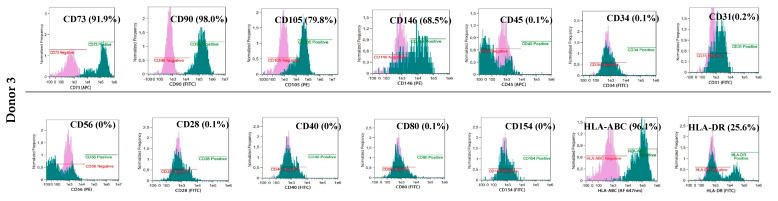
Phenotype of MSCs isolated from the donor’s bone marrow and delivered intramuscularly to DMD patients. Isolated BM-MSCs show the basic naïve MSC phenotype CD73+/CD90+/CD105+, proangiogenic properties CD146+, expression of HLA-class I antigens, lack or low expression of HLA-DR antigens, lack of co-stimulatory molecules CD28, CD40, CD80, CD154 and lack of the hematopoietic phenotype CD34, CD45.

**Figure 5:**
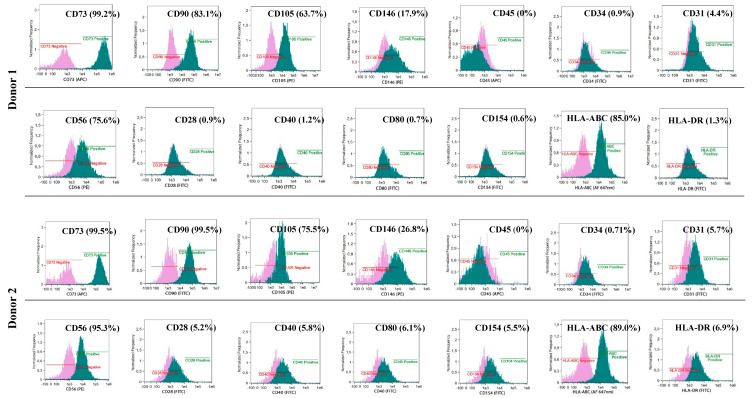

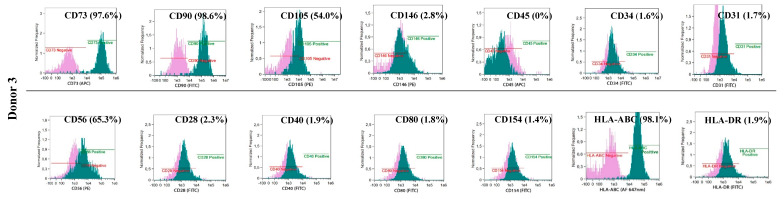
Phenotype of myogenic stem/progenitor cells isolated from the donor’s skeletal muscles. Isolated myogenic stem/progenitor cells show the basic naïve MSC phenotype CD73+/CD90+/CD105+, proangiogenic properties CD146+, expression of HLA-class I antigens, low expression of HLA-DR antigens, low number of cells expressing co-stimulatory molecules CD28, CD40, CD80, CD154 and lack of the hematopoietic phenotype CD45. Myogenic stem/progenitor cells express the specific phenotype CD56.

BM-derived and myogenic adherent cells exhibited a similar phenotype CD90+, CD73+ and CD105+ characteristic for naïve MSCs (85%–99% of adherent cells) (Figure 4 and Figure 5). All cultured cells derived from BM and myogenic stem/progenitor cells were negative for the hematopoietic marker CD34 and CD45. The BM-MSCs and myogenic stem/progenitor cells expressed HLA class I antigens with frequencies from 85% to 99%. The expression of HLA-DR antigens on BM-MSCs varied and was assessed at 0.14% (Donor 1), 2.2% (Donor 2) and 25.6% (Donor 3), whereas for the myogenic stem/progenitor cells, the values were assessed at 1.3% (Donor 1), 6.9% (Donor 2), and 1.9% (Donor 3). BM-MSCs from all donors were negative for co-stimulatory molecules, which was proved by a lack of expression of CD28, CD40, CD80, and CD154 (Figure 4). Co-stimulatory molecules on myogenic stem/progenitor cells were expressed by between 0.6% and 1.2% of cells (Donor 1), by between 5.2% and 6.1% of cells (Donor 2) and by between 1.4% and 2.3% of cells (Donor 3) (Figure 5).

The proangiogenic properties of the BM-MSCs were confirmed by the expression of CD146 in 26.8% (Donor 1), 72.1% (Donor 2) and 68.5% (Donor 3) of the adherent cells (Figure 4); for the myogenic progenitors, the values were 17.9% (Donor 1), 26.8% (Donor 2), and 22.8% (Donor 3) of the adherent cells expressing CD146 (Figure 5).

### 3.3. Distinct Phenotype of Myogenic Stem/Progenitor Cells

Myogenic stem/progenitor cells (but not BM-MSCs) additionally expressed CD56 in 75.6% (Donor 1), 95.3% (Donor 2) and 65.3% (Donor 3) of adherent cells (Figure 5). This observation suggests that these populations of stem/progenitor cells were muscle-specific.

### 3.4. Spontaneous Fusion of BM-MSCs and Myogenic Stem/Progenitor Cells In Vitro

**Figure 6:**
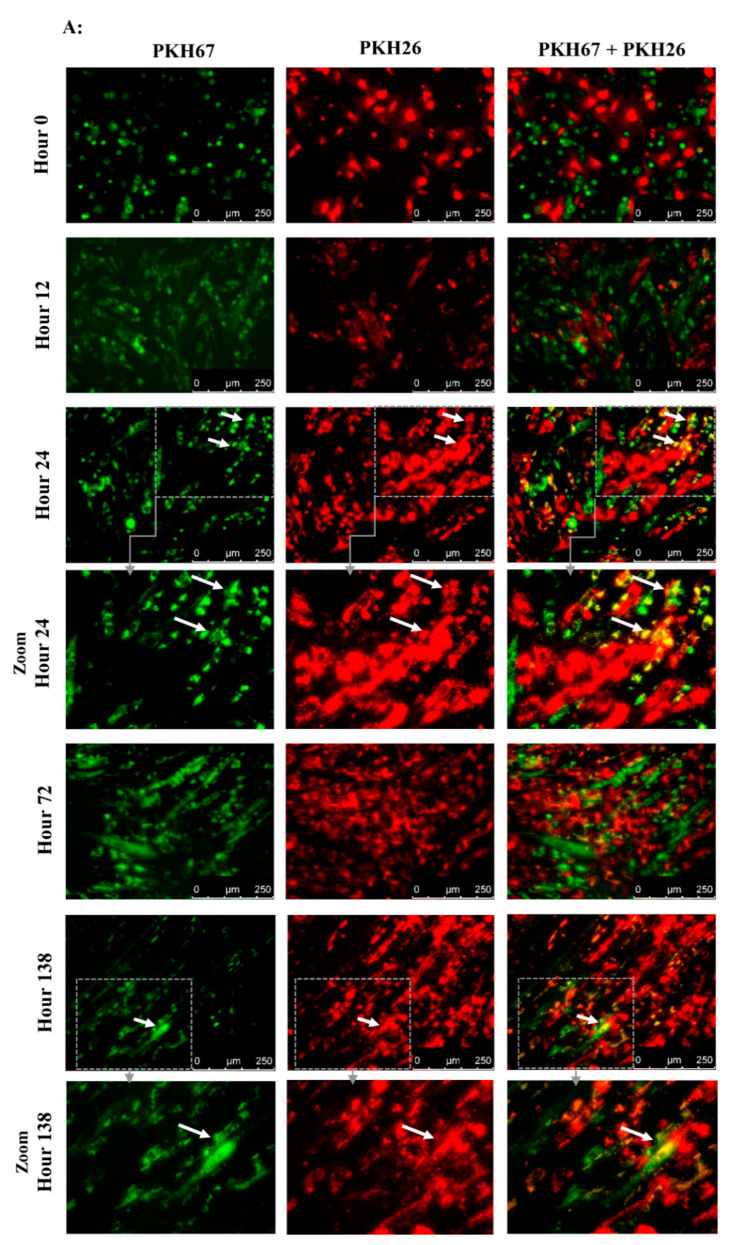

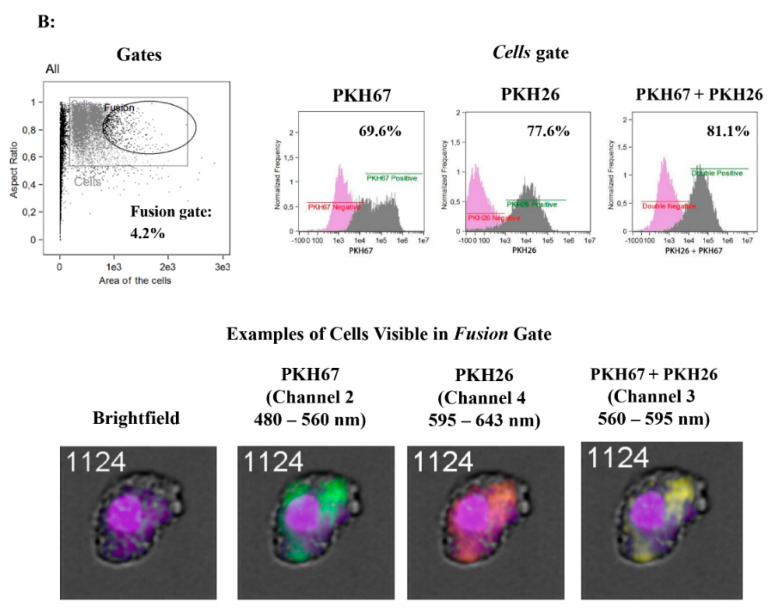
Spontaneous fusion between BM-MSCs and SM-SPCs *in vitro.* A representative illustration of co-cultured cells obtained from Donor 1. (**A**) Immunofluorescence staining with PKH26 (red) for BM-MSCs and PKH67 (green) for SM-SPC revealed fused cells between SM-SPCs (green multinucleated cells) and between BM-MSCs and SM-SPCs (yellow/orange) as early as 24 h after the co-culture was started. The areas limited by grey lines are enlarged by zoom. (**B**) To confirm the spontaneous fusion between BM-MSCs and SM-SPCs, the mixed co-culture was detached from the culture plate on day 6, and single cells were analysed with flow cytometry to assess the presence of cells revealing double-merged fluorescence signals. Flow cytometry analysis showed cell populations with fluorescence emission in the 480–560 nm spectrum (Channel 2) characteristic for PKH67, the 595–643 nm spectrum (Channel 4) characteristic for PKH26, and the 560–595 nm spectrum (Channel 3), which suggests the immersion of two dyes with each other.

A spontaneous fusion between BM-MSCs and myogenic progenitors was observed as early as 24 h after the start of the in vitro co-culture (Figure 6A). After 24 h, the first structures resembling myotubes formed exclusively from SM-SPCs (represented by multinucleated green cells) and were clearly visible 72 h after the co-culture was started. At the same time, the structures formed from BM-MSCs and SM-SPCs (represented by yellow/orange stained cells) were visible within the co-cultured cells. However, during the following days, the co-cultures expanded extensively, and it was difficult to distinguish the numerous fused cells from those which were growing separately on a multilayer base (Figure 6A).

To confirm the spontaneous fusion between the co-cultured BM-MSCs and SM-SPCs, the mixed co-cultures were detached from the culture plate on day 6, and single cells were analysed using flow cytometry to assess the presence of cells revealing double-merged fluorescence signals. The flow cytometry analysis showed cell populations with a fluorescence emission in the 480–560 nm spectrum (Channel 2) characteristic for PKH67, the 595–643 nm spectrum (Channel 4) characteristic for PKH26 and the 560–595 nm spectrum (Channel 3), which suggests the immersion of two dyes with each other. On day 6, the co-culture of BM-MSCs and SM-SPCs from Donor 1 and Donor 2 revealed a fluorescence emission in Channel 3 in the population specific for double-positive cells. These cells were characterised by a specific morphology with at least double-cell nuclei and a strong fluorescence in the three examined channels (Figure 6B). Co-culture of cells from Donor 3 was not performed due to a limited number of BM-MSCs, and priority was given to the delivery of these cells to Patient 3 for the planned cellular treatment.

### 3.5. Histological and mRNA Analysis of Muscle Biopsies

**Figure 7:**
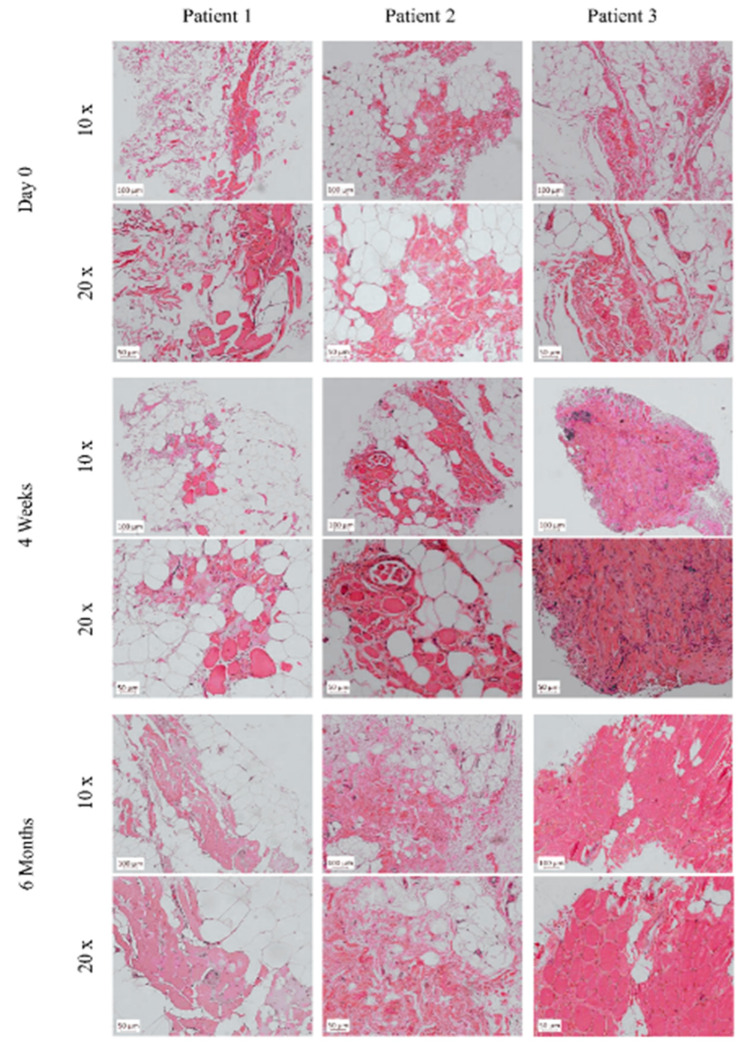
Histopathological changes of skeletal muscles in DMD patients. Muscle biopsies taken from the patients on day 0, before cellular therapy, revealed the predominance of fat or connective tissue; these changes corresponded to Grade 4 according to the Muntoni scale [20]. In the biopsy taken from Patient 1 on day 0, focal muscle fibres were surrounded by fat and connective tissue. However, during the follow-up period after six months, more numerous muscle fibres in the cell-grafted area were present. In the biopsy from Patient 2, on day 0, abundant substitutions by fat and connective tissue were evident. Four weeks after cellular therapy, focal myofibres were detected at the cell-grafted site, however, after six months, only the fibrotic and fat tissues were detected in the examined biopsies. Biopsy taken from patient 3 also revealed only fat and connective tissue at day 0. Four weeks after the cell transplantation, muscle tissue with disrupted muscle fascicles, marked fibrotic tissue and abundant inflammatory infiltrates was visible. Six months after cellular therapy, inflammatory infiltrates between the muscle fibres were very moderate compared to week 4.

**Figure 8:**
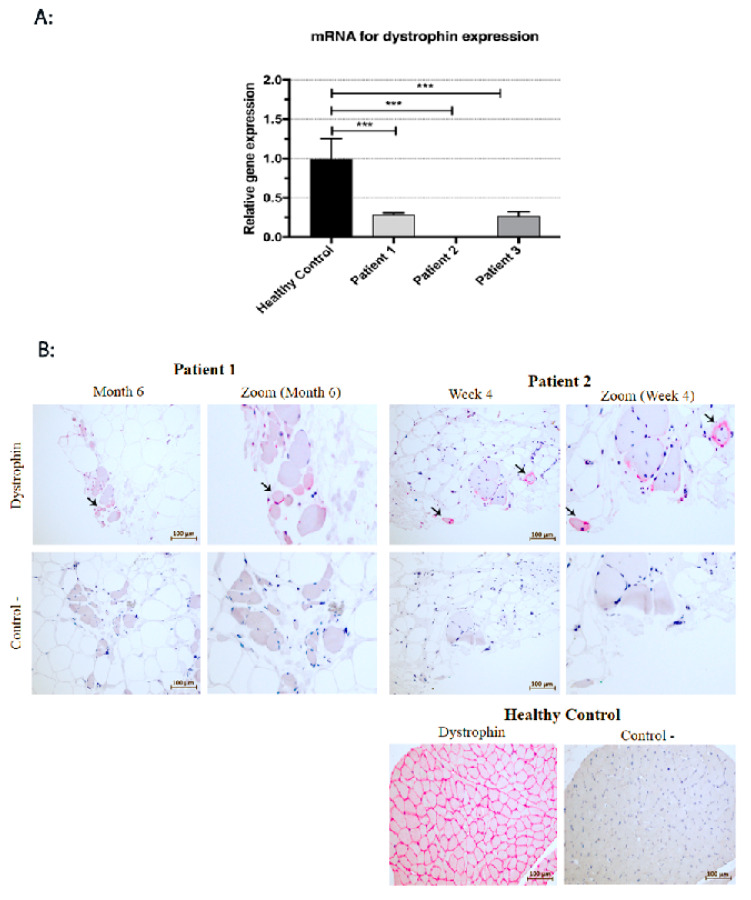
Dystrophin expression at mRNA and protein level. (**A**) Four weeks after the cellular therapy, mRNA for *dystrophin* gene expression in Patient 1 and Patient 3 was assessed at 20% (RQ = 0.206) and at 27% (RQ = 0.265), respectively, of healthy controls (*** *p* < 0.001). (**B**) In Patient 1, six months after the co-transplantation of BM-MSCs and myogenic stem/progenitor cells, about 15% of small myofibres expressed dystrophin (arrow). Myofibres expressing dystrophin (arrow) were present in the tissue samples taken from Patent 2 at 4 weeks after cellular therapy (EnVision^TM^ FLEX/HRP detection system staining with HRP magenta substrate chromogen).

Muscle biopsies taken from the patients on day 0, before the cell transplantation, revealed an image corresponding to Grade 4, as introduced by the Muntoni Group [20] (Figure 7). Grade 4 in a DMD muscle is diagnosed when more than 50% of the analysed muscle biopsy has been replaced by fat or connective tissue. In the biopsy taken from Patient 1 on day 0, the focal muscle fibres were surrounded by fat and connective tissue. However, during the follow-up period six months after, numerous muscle fibres in the cell-grafted area were present, although adipose tissue and focal fibrosis were still visible. mRNA for dystrophin gene expression was assessed at a level of 20% compared to healthy controls (RQ = 0.206; *p* < 0.001) (Figure 8A). Around 15% of the myofibres expressed dystrophin six months after the BM-MSC and SM-SPC delivery (Figure 8B). In the biopsy from Patient 2, on day 0, abundant substitutions by fat and connective tissue were evident. Four weeks after the cellular therapy, focal myofibres were detected at the cell-grafted site, and single small myogenic cells expressed dystrophin (Figure 8B). Due to a low level of RNA (2–3 ng/mL) isolated from the tissue sample, mRNA for the dystrophin gene was undetectable in the examined tissue taken from Patient 2. However, six months after the therapy, only the fibrotic and fat tissues were present in the examined biopsies (Figure 7). The biopsy taken from Patient 3 also revealed only fat and connective tissue on day 0, and we assumed that the very small biopsy taken on day 0 was insufficient for an adequate assessment. This assumption was confirmed by our observation in the biopsy taken four weeks after the cell transplantation, which revealed muscle tissue with a disruption of muscle fascicles, marked fibrotic tissue and abundant inflammatory infiltrates (Figure 7) corresponding to Grade 3 of the histopathological assessment in DMD [20]. mRNA for the dystrophin gene expression in Patient 3 was assessed at 27% of the corresponding level in healthy controls (RQ = 0.265; *p* < 0.001) (Figure 8A). Six months after cellular therapy, numerous myofibres were visible in the examined biopsy from Patient 3. However, the inflammatory infiltrates were moderate compared to week 4 after the cellular therapy (Figure 7). Dystrophin at the protein level was undetectable during the follow-up period.

### 3.6. Serum Levels of Cytokines and Growth Factors

**Figure 9:**
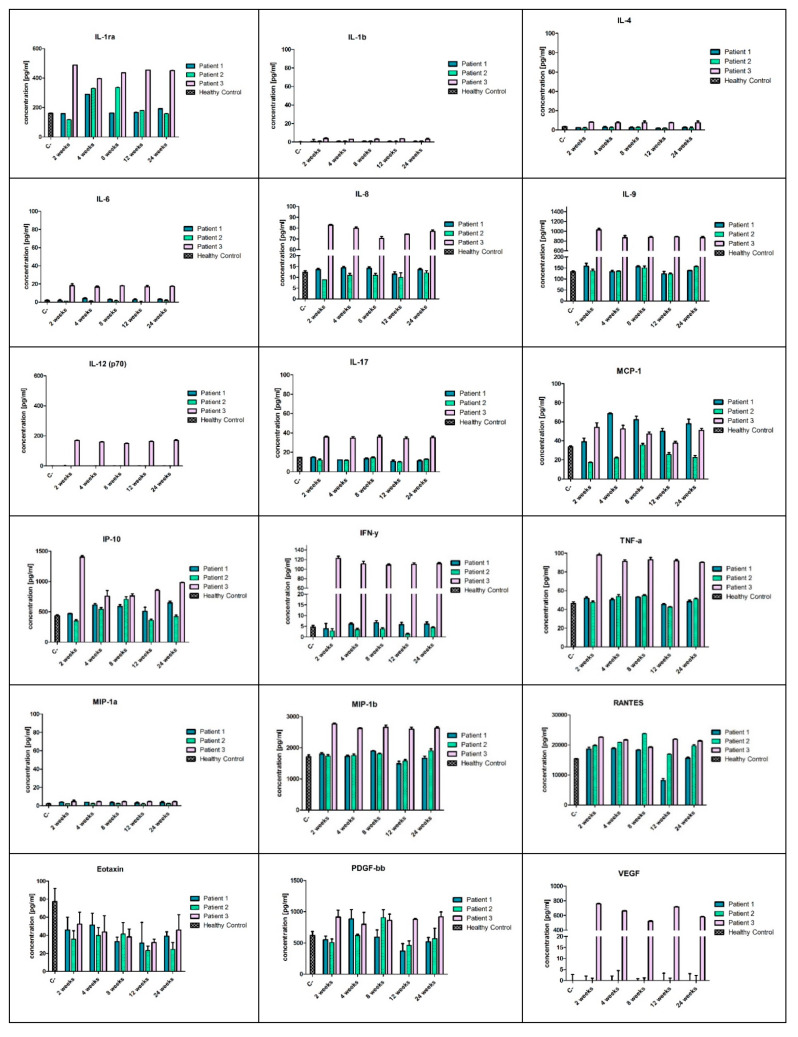
A panel of cytokines, chemokines and growth factors with the multiplex ELISA assay. The cytokine levels in the sera of DMD patients were assessed with reference to the serum levels of healthy controls. Most of the proinflammatory cytokines and chemokines, including IL-1β, IL-6, IL-8, IL-9, IL-12, IL-17, IFNγ, TNFα, MIP-1α and MIP-1β in Patient 1 and 2, were at the levels of healthy controls or slightly below it, such as IFNγ in Patient 2. All analysed proinflammatory cytokines and chemokines were significantly upregulated during the follow-up period in Patient 3.

Chronic inflammation is a hallmark of DMD progress. In this study, we analysed a panel of cytokines and growth factors using a multiplex ELISA assay starting from two weeks after the cell transplantation up to six months of follow-up. The cytokine levels in the sera of the DMD patients were assessed with reference to the serum levels of healthy controls. Overall, most of the proinflammatory cytokines and chemokines, including IL-1β, IL-6, IL-8, IL-9, IL-12, IL-17, IFNγ, TNFα, MIP-1α, and MIP-1β in Patient 1 and Patient 2, were at the healthy control level or slightly below it, such as IFNγ in Patient 2 (Figure 9). All analysed proinflammatory cytokines and chemokines were significantly upregulated during the follow-up period in Patient 3 (Figure 9). The level of IL-1Ra varied during the observation period, and at four weeks after cellular therapy, it was elevated in all patients, then returned to the control level in Patients 1 and 2. MCP-1 also differed between the patients, and the highest concentration was assessed in Patients 1 and 3 during the follow-up period, whereas in Patient 2, the MCP-1 level was lower compared to control. RANTES level increased in all patients (except Patient 1 after three and six months of observation) compared to control. Eotaxin concentration decreased in all patients during the follow-up period. Details of analysis are presented in Figure 9.

## 4. Discussion

Cellular therapies in DMD patients have a long history. Over 25 years ago, adoptive therapy with myoblasts isolated from the skeletal muscles of healthy donors was introduced to clinical practice [7,8,9]. Unsatisfactory results of the treatment with myoblasts were explained with their decreased migratory and limited proliferative potential in vivo [11,12,21].

Our hypothesis about the beneficial effect of cellular therapy when co-transplanting BM-MSCs and myogenic stem/progenitors using the ‘high-density injection’ protocol was verified in three cases of DMD patients at different stages of the disease and with different types of the gene mutation. Although it is difficult to compare the effect of the therapy in the group, certain common features of the positive clinical response can still be established. In this project, the patients were observed up to six months only, and in two patients (Patient 1 did not cooperate in the EMG assessment), the functional assessment performed by an EMG analysis (after six months) revealed an improvement in motor unit parameters, especially in the area and size index, compared to the initial test before the BM-MSC and SM-SPC delivery. Dystrophin expression at mRNA and protein level was only observed in one patient, who received the therapeutic cells into the right gastrocnemius. This patient had a better functional outcome in the right limb injected with the BM-MSCs and SM-SPCs. However, mRNA for dystrophin was detectable in two patients with the histological hallmark of muscle fibres. This observation may substantiate the use of the ‘high-density injection’ protocol and the local intramuscular administration of cells, as proposed by Skuk et al. in previous studies with myoblast therapy in DMD patients [14]. The additional type of stem cells (BM-MSCs), used in our protocol, may support the pro-regenerative potential of myogenic stem/progenitor cells thanks to their trophic, paracrine, and immunomodulatory activity. 

Tacrolimus used for immunosuppression was chosen in our procedure with respect to the previous protocol introduced by Skuk et al. [11]. The authors emphasised the advantage of tacrolimus over cyclosporine because earlier studies documented that cyclosporine inhibited the fusion and differentiation of myogenic cells and induced apoptosis when myogenic precursors started to differentiate [11,22]. Our immunosuppressive protocol revealed certain advantages over the others and was stopped three weeks after the combined BM-MSC and myogenic stem/progenitor cell co-implantation, and no adverse effects were observed when tacrolimus was withdrawn. This observation supports our hypothesis that MSCs derived from bone marrow may play a double role in the applied scenario. One role is the direct fusion of BM-MSCs and/or SM-SPCs, as confirmed in our study with an ex vivo analysis. A substantial number of myogenic stem/progenitor cells fused with each other or fused with BM-MSCs and formed myotubes. However, we are aware that the number of cells fused in vitro does not reflect the ability of these cells to fuse in vivo in DMD patients with the same efficacy due to the dystrophic microenvironment associated with proinflammatory cytokine activity [17,23]. We also suggest that the second pro-regenerative capacity of BM-MSC involves the immunomodulatory properties that can change the dystrophic microenvironment through the secretion of immunosuppressive cytokines, thus augmenting the regenerative potential of SM-SPCs in direct cell-to-cell contact and/or through the secretion of the trophic factors that control muscle progenitor proliferation and myogenic differentiation [16]. Our hypothesis can also be confirmed by the very recent clinical studies on the effects of allogeneic MSCs derived from Wharton jelly on the functioning of skeletal muscles based on the ability of MSCs to fuse with DMD muscles or to induce organelle nanotransfer [24]. Some authors explained this phenomenon through the activity of exosomes, acting as a cargo of miRNA from the transplanted MSCs, which are able to fuse with the plasma membrane of the dystrophic muscles to modulate gene expression and may promote muscle regeneration [24,25].

Moreover, MSCs derived from bone marrow may support the inhibition of the inflammatory process not only at the site of stem cell delivery [26]. Muscle degeneration in DMD is accompanied by a chronic inflammation associated with the overexpression of proinflammatory cytokines, such as TNF-α, IL-1 and IL-6 in the skeletal muscles and in the blood serum [17,23]. Inflammatory infiltrates consisting of CD4 and CD8 T lymphocytes, neutrophils, and macrophages with the pro-inflammatory phenotype M1 and immunoregulatory phenotype M2 may participate in cytokine production, resulting in a cytolytic and cytotoxic effect on the muscle fibres [27,28,29]. MSCs have the ability to modify the inflammatory M1 type of macrophages (pro-inflammatory, anti-angiogenic and tissue growth inhibitors) into the M2 phenotype (anti-inflammatory, pro-remodelling, and tissue healing), which is favourable for skeletal muscle regeneration [16,30]. Our protocol of the co-transplantation of BM-MSCs and SM-SPCs confirmed the immunosuppressive properties of MSCs, which was reflected by the lack of observed systemic and/or local inflammation, especially in Patients 1 and 2. In both patients, the concentrations of proinflammatory cytokines, such as IL-1β, IL-6, IL-8, IL-9, IL-12, IL-17, IFN-γ, TNF-α, MIP-1α, and MIP-1β, were at levels comparable to healthy controls. Additionally, the anti-inflammatory effect of BM-MSCs was confirmed in our patients by an increased level of the interleukin 1 receptor antagonist (IL-1Ra), a naturally-occurring cytokine that inhibits the binding of IL-1β to IL-1R. Muscle inflammation in DMD is primarily mediated by the IL-1β pathway, and the inhibition of its activity may potentially decrease inflammatory effects (the anti-inflammatory properties of IL-Ra were proved in the mdx mouse model through a functional improvement of the dystrophic muscle) [31]. Moreover, we did not observe any clinical signs of systemic inflammation, such as fever or chill, in Patients 1 and 2. Modulation of inflammation is beneficial in DMD, and MSCs are considered as anti-inflammatories for the treatment of DMD [32].

The number and ratio of the transplanted BM-MSCs and SM-SPCs likely also affected the outcome of the cell delivery. In Patients 1 and 2, the ratio of BM-MSCs and SM-SPCs was similar and amounted to approximately 1:2. In Patient 3, who showed a high level of systemic proinflammatory cytokines, the total number of stem/progenitor cells was similar to the other patients; however, the ratio of BM-MSCs and SM-SPCs differed and amounted to 1:10. The reason was a low proliferative potential of BM-MSCs (or its intrinsic defect) from Donor 3 during the first phase of cell expansion. In this protocol, we designed the prospective delivery of isolated and expanded BM-MSCs with SM-SPCs, and we did not plan to use cryopreserved cells. For Patient 3, we used the BM-MSCs in the amount obtained when the SM-SPCs were confluent and sufficient in number. We assume that the lower number of BM-MSCs may have also caused the accumulation of inflammatory infiltrates, impaired fusion of SM-SPCs and a subsequent lack of dystrophin expression in the examined biopsies in Patient 3, but this assumption needs to be explored in future studies. 

The high level of proinflammatory cytokines in the serum of Patient 3 may be explained by the recent report that inflammation in DMD patients is associated with an intense progression of muscle damage [17]. The patients with better muscle function revealed the highest serum levels of proinflammatory cytokines, such as IL-1 and TNF-α, and creatine kinase, compared to patients with weak muscle function. These studies may also support this notion, since Patient 3, who remained independently mobile, showed significantly higher levels of proinflammatory cytokines and creatine kinase compared to Patients 1 and 2, who were both wheelchair-dependent. However, in the oldest patient (Patient 2, aged 22 years) with the poorest muscle function, some of cytokines, such as IFN-γ and MCP-1, were below the control levels; this observation may also reflect the severity of muscle damage. Systemic inflammation may exhaust the degeneration and regeneration cycles of the muscle fibres, leading to the deposition of connective and adipose tissues, which is a histological hallmark of the disease progression [20]. Excessive accumulation of fat and fibrotic tissues was observed in muscle biopsies drawn from Patients 1 and 2, which was associated with the loss of muscle function documented by the poor functional status of both patients. Moreover, an important contribution to the inflammation is the fact that the damaged muscle fibres were themselves able to produce and secrete cytokines [33]. Studies on the expression of IL-17 mRNA in muscle biopsies from DMD patients showed correlations between mRNA levels for IL-17, TNF-α, and MCP-1 in dystrophic skeletal muscles. Nevertheless, the systemic levels of cytokines were not assessed in that study [34]. Thus, extrapolating from the previous studies, we may conclude that increased levels of systemic pro-inflammatory cytokines were predominantly observed at the early stages of the disease in younger DMD patients, who still showed muscle function [17].

The favourable effect of the therapy with BM-MSCs and SM-SPCs was also confirmed by lowered creatine kinase levels, considered as a biomarker of muscle damage. Creatine kinase level is known to decrease as the disease progresses, especially in older patients [17]. This observation may be further confirmed through Patient 2, who displayed the lowest level of creatine kinase before the cellular therapy, and a similar level was observed at the end of our observations. In Patients 1 and 3, the creatine kinase level was initially much higher compared to Patient 2 and decreased significantly during the follow-up period. A decrease in the serum creatine kinase level in DMD patients after therapy with MSCs derived from Wharton jelly (administered intramuscularly and intra-arterially) was also confirmed in a recent clinical trial [24]. The beneficial effect of intramuscular MSC delivery of lowering the creatine kinase level has recently been reported in a study on the intramuscular transplantation of placenta-derived MSCs in mdx mouse [35]. Another study on the experimental mdx mouse model has also confirmed that the creatine kinase level and plasma cytokine levels, especially with respect to TNFα, INFγ, IL-6, and TGFβ, were useful biomarkers for the assessment of the disease progression and the response to cellular therapy [36].

Our protocol of BM-MSC and myogenic stem/progenitor cell co-transplantation (obtained from the same related donor) did not generate any adverse effects after the local intramuscular high-density microinjections. We did not observe any complications after the intramuscular cell delivery, and the high level of proinflammatory cytokines in the serum of Patient 3 was more likely associated with the early progressive stage of the disease than with the instrumentation of the cellular therapy itself. This was also confirmed by a lack of upregulated inflammatory cytokines in the sera of Patients 1 and 2 after the BM-MSC and SM-SPC delivery. Moreover, the immunosuppressive properties of MSCs may help to reduce immune activities towards muscle stem/progenitor cells in allogenic conditions [16].

The protocol of cellular therapy used in this study involving a local administration of BM-MSCs and SM-SPCs also had certain limitations, i.e., the low number of patients, the variable doses of the delivered cells and their proportion, as well as the genetic background and stage of the disease. Further clinical studies including more DMD patients at an early stage of the disease (with similar clinical manifestations) and possibly similar genetic mutations will be necessary to confirm our hypothesis. 

## 5. Conclusions

Stem/progenitor cells generated from BM and skeletal muscles expressed the phenotypic characteristics of naïve MSCs, did not express the immunogenic phenotype, and were able to generate spontaneous cell fusion under in vitro conditions.

The development of cell-based therapies involving the delivery of two defined populations of normal stem/progenitor cells (BM-MSCs and SM-SPCs) may be a promising tool for the treatment of muscular dystrophies. To the best our knowledge, this is the first clinical observation on the therapeutic effects of BM-MSCs and SM-SPCs in patients with DMD. The obtained results suggest that response to cellular therapy is case-dependent and is associated with the pre-treatment clinical stage of disease. However, the course of the disease can be ameliorated through a lowered creatine kinase level and an increase of IL-1Ra with anti-inflammatory properties. Moreover, with high likelihood, the fusion between transplanted cell populations and the degenerating DMD skeletal muscle cells of the recipient facilitates skeletal muscle recovery. However, further research is required to optimise the timing and dosing of stem/progenitor cell delivery.

DMD is a genetic disorder, and with the present state of knowledge, it is unlikely that a patient will fully recuperate. However, advanced research on cell therapy in DMD and the results presented in this paper suggest that it is possible to inhibit the progression of the disease in certain vital regions of the body, ameliorating its course by improving the muscle function of the patients, since there is no other option for the treatment of patients with DMD.

## Figures and Tables

**Table 1:** Patient and donor characteristics.

	Patient 1	Patient 2	Patient 3
Patient age	11	22	12
Donor age	37	17	57
Dystrophin gene mutation	Deletion of exons 8–22 (out of frame)	Duplication of exon 2 (out of frame)	Deletion of exons 51–57
Disorder	*De novo*	Inherited	Inherited
Functional status	Wheelchair from age 9	Wheelchair from age 12	Still ambulant, needs support when walking
HLA–Patient	A 02,03; B 41,07; C 07,17; DR 13,15	A 02,03; B 07,13; C 06,07; DR 7,15	A 23,25; B 15,18; C 07,12; DR 11,15
HLA–Donor	A 01,03; B 08,07; C 07,-; DR 03,15	A 01,03; B 37,07; C 06,07; DR 11,15	A 02,25, B 18,50, C 06,12; DR 11,15
Donor	father	sister	father
Prior treatment	No	No	No
Tacrolimus blood level	7.8 ng/mL	NA	4.2 ng/mL
Total number of co-transplanted BM-MSCs and SM-SPC cells	820 × 10^6^	740 × 10^6^	700 × 10^6^
Injected area	Right gastrocnemius (18 cm^2^)	Right biceps brachii (10 cm^2^)	Right biceps brachii (9 cm^2^)

BM-MSC—bone marrow mesenchymal stem cells; SM-SPC—skeletal muscle stem/progenitor cells.

**Table 2:** Characteristics of BM-MSCs and myogenic stem/progenitor cells.

	Donor 1	Donor 2	Donor 3
Bone marrow aspiration	50 mL	50 mL	50 mL
Total NCC	138 × 10^7^	180 × 10^7^	81 × 10^7^
Number of passages	2	3	2
Total production of BM-MSCs	325 × 10^6^	228 × 10^6^	75 × 10^6^
Total production of myogenic stem/progenitor cells	520 × 10^6^	520 × 10^6^	627 × 10^6^
BM-MSC purity (%) (CD73+/CD90+/CD105+)	97.9/99.5/75.5%	97.8/99.4/78.5%	91.9/98.0/79.8%
SM-SPC (%) (CD73+/CD90+/CD105+)	99.2/83.1/63.7%	99.5/99.5/75.5%	97.6/98.6/54.0%
Myoblasts CD56+ (%)	75.5%	95.3%	65.3%
Microbial contamination	negative	negative	negative

NCC—Nucleated cell concentrate; BM-MSC—bone marrow mesenchymal stem cells; SM-SPC—skeletal muscle stem/progenitor cells.

**Table 3:** Primers used for mRNA dystrophin expression.

Species	Gene	Id	Product (bp)	R2	Efficiency (%)	Cq	Cm (°C)
*H. Sapiens*	*DMD*	qHsaCID0010707	129	0.9998	101	21.96	82.5
*H. Sapiens*	*ACTB*	qHsaCED0036269	62	0.9939	103	15.155	82
*H. Sapiens*	*GAPDH*	qHsaCED0038674	117	0.999	97	13.2	86
*H. Sapiens*	*TBP*	qHsaCID0007122	120	0.9997	101	21.62	83

**Table 4:** Electromyography examination of Patient 2; MUP parameters of the biceps brachii muscle.

MUP* Parameters	Time in Relation to BM-MSC and SM-SPC Delivery	
	Before delivery	6 months after delivery
Duration (ms)	10.0	9.9
Amplitude (µV)	1816	1591
Area (µV ms)	3035	3139
Size index	2.12	2.25

*MUP—motor unit parameter.

**Table 5:** Electromyographyexamination of Patient 3; MUP parameters of the biceps brachii muscle.

MUP* Parameters	Time in Relation to BM-MSC and SM-SPC Delivery	
	Before therapy	6 months after therapy
Duration (ms)	4.8	7.3
Amplitude (µV)	903	1239
Area (µV ms)	568	1728
Size index	0.35	1.2

*MUP—motor unit parameter.
